# Mechanism of Cisplatin-Induced Cytotoxicity Is Correlated to Impaired Metabolism Due to Mitochondrial ROS Generation

**DOI:** 10.1371/journal.pone.0135083

**Published:** 2015-08-06

**Authors:** Yong-Min Choi, Han-Kyul Kim, Wooyoung Shim, Muhammad Ayaz Anwar, Ji-Woong Kwon, Hyuk-Kwon Kwon, Hyung Joong Kim, Hyobin Jeong, Hwan Myung Kim, Daehee Hwang, Hyung Sik Kim, Sangdun Choi

**Affiliations:** 1 Department of Molecular Science and Technology, Ajou University, Suwon, 443–749, Korea; 2 Division of Energy Systems Research, Ajou University, Suwon, 443–749, Korea; 3 School of Interdisciplinary Bioscience and Bioengineering, POSTECH, Pohang, 790–784, Korea; 4 School of Pharmacy, Sungkyunkwan University, Suwon, 440–746, Korea; National Institutes of Health, UNITED STATES

## Abstract

The chemotherapeutic use of cisplatin is limited by its severe side effects. In this study, by conducting different omics data analyses, we demonstrated that cisplatin induces cell death in a proximal tubular cell line by suppressing glycolysis- and tricarboxylic acid (TCA)/mitochondria-related genes. Furthermore, analysis of the urine from cisplatin-treated rats revealed the lower expression levels of enzymes involved in glycolysis, TCA cycle, and genes related to mitochondrial stability and confirmed the cisplatin-related metabolic abnormalities. Additionally, an increase in the level of p53, which directly inhibits glycolysis, has been observed. Inhibition of p53 restored glycolysis and significantly reduced the rate of cell death at 24 h and 48 h due to p53 inhibition. The foremost reason of cisplatin-related cytotoxicity has been correlated to the generation of mitochondrial reactive oxygen species (ROS) that influence multiple pathways. Abnormalities in these pathways resulted in the collapse of mitochondrial energy production, which in turn sensitized the cells to death. The quenching of ROS led to the amelioration of the affected pathways. Considering these observations, it can be concluded that there is a significant correlation between cisplatin and metabolic dysfunctions involving mROS as the major player.

## Introduction

Platinum-derived chemotherapeutic agents, including the parent compound cis-diamminedichloroplatinum (II) (cisplatin), are the principal therapeutic agents to treat various cancers, including ovarian, testicular, and bladder cancer [1,2]. However, the therapeutic use of cisplatin is limited by its irreversible side effects, including transcription inhibition, cell cycle arrest, generation of reactive oxygen species (ROS), and apoptosis [3].

In recent years, several studies demonstrated that cisplatin-induced cytotoxicity is closely related to increased ROS generation [4,5] ROS is a by-product of the normal cellular metabolism; however, excessive amounts can cause detrimental effects. In particular, ROS is an important factor in the apoptosis pathway; increased ROS generation alters the mitochondrial membrane potential (MMP) and damages the respiratory chain, which ultimately triggers the apoptotic process [6,7]. Previous reports demonstrated that compounds that interfere with ROS generation could inhibit cisplatin-induced apoptosis; correlating cisplatin toxicity to the activation of oxidative stress pathways [2].

P53 is an important anticancer gene that is frequently mutated in cancers. It has been reported that cisplatin induces the expression of p53, which plays diverse roles, particularly in regulating glycolysis genes [8–11]. Down-regulation of glycolysis genes when p53 is over-expressed leads to apoptosis. Moreover, energy status of cell is the main determining factor in cell survival or cell death. Severe reduction of adenosine triphosphate (ATP) level initiates necrosis because apoptosis-dependent pathways require ATP [12]. Therefore, in cisplatin-induced cytotoxicity, energy depletion of cells is a crucial factor that is being perpetuated by disrupting metabolic pathways.

Cisplatin rapidly accumulates in mitochondria and deteriorates the mitochondrial structure and metabolic function [13]. This leads to significant changes in the metabolites level related to the tricarboxylic acid cycle (TCA cycle) and glycolysis pathway [14,15]. However, the precise mechanism of cisplatin-induced metabolic toxicity remains elusive.

The following study aimed to elucidate the metabolic dysfunctions in a proximal tubular cell line (human kidney 2; HK-2) following treatment with cisplatin. A systems biology approach was employed to investigate the role of mitochondrial dysfunction and abnormal metabolic pathways in cisplatin-induced kidney injury. Different techniques have been used to delineate physiological and morphological anomalies. Finally, cytotoxic events induced by cisplatin were evaluated in HK-2 cells with and without various inhibitors. Moreover, we established a network that describes the mechanisms of cisplatin-induced metabolic toxicity in HK-2 cells.

## Materials and Methods

### Cell culture

The cell line HK-2 (ATCC # CRL-2190; Manassas, VA, USA) was purchased from the American Type Culture Collection. This cell line has been widely accepted as the standard cell line for normal renal tubular epithelium [16,17]. Cells were grown in keratinocyte-serum-free medium supplemented with l-glutamine, epidermal growth factor, and bovine pituitary extract (Gibco, Carlsbad, CA, USA) and maintained at 37°C under 5% CO_2_.

### Cell proliferation assay (MTS assay)

Cell proliferation was measured colorimetrically using 3-(4, 5-dimethylthiazol-2-yl)-5-(3-carboxymethoxyphenyl)-2-(4-sulfophenyl)-2H-tetrazolium (MTS) solution (Cell Titer 96H; Promega, Madison, WI, USA). HK-2 cells were grown in 96-well plates at a density of 10,000 cells per well. After treatment for the indicated times, MTS solution (10 μL/well) was added to the wells, the cells were incubated for 3 h at 37°C, and absorbance was measured using the VERSA microplate reader (Turner Biosystems, Sunnyvale, CA, USA).

### Lactate dehydrogenase assay

HK-2 cells were seeded in a 96-well plate and grown at 37°C and 5% CO_2_ overnight. Cell viability was determined at different times using the CytoTox-Glo cytotoxicity assay (Promega). Briefly, 50 μL of CytoTox-Glo cytotoxicity assay reagent was added to each well and the plate was incubated at room temperature for 15 min. The luminescence intensity was measured using the VERITAS microplate luminometer (Turner Biosystems).

### Lactate measurement in glycolysis and NAC treatment

Lactate concentration was measured using the Lactate Colorimetric Assay Kit (Abcam, Cambridge, MA, USA). Equal number of cells were suspend in Lactate Assay Buffer and all experimental procedure was performed according to the instruction of the manufacturer. Experiment was repeated three times and absorbance was measured using the VERSA microplate reader (Turner Biosystems). For p53 inhibition, 20 μM pifithrin-α (PFT-α) (Sigma-Aldrich, MO, USA) was treated for 1 h before the cisplatin treatment and quantifications were made at indicated time points. For N-acetylcysteine (NAC) (Sigma-Aldrich), which is a powerful antioxidant, 10 μM NAC was pretreated for 1 h and then cisplatin was applied. All experiments related to PFT-α and NAC followed the same treatment process.

### ATP quantification assay

ATP concentration was quantitatively determined using an ATP determination kit (Invitrogen, Carlsbad, CA, USA) according to the manufacturer’s instructions. Cells were resuspended in 1× reaction buffer. Then, 10 μL of the cell suspension was transferred to a new 96-well plate and 90 μL of standard reaction solution was added. The plate was incubated for 15 min at room temperature. ATP was quantified using the VERITAS microplate luminometer (Turner Biosystems) and the LAS-1000 system (FUJIFILM, Tokyo, Japan).

### Glucose assay

The intracellular glucose concentration was measured using the Amplex Red glucose/glucose oxidase assay kit (Invitrogen) according to the instructions of the manufacturer. Cells were lysed using 1× reaction buffer. Then, 100 μL of the lysate was resuspended in 400 μL of 1× reaction buffer. Next, 50 μL of the glucose working solution was added to 50 μL of cell lysate in a 96-well plate. The mixture was incubated in the dark for 30 min at room temperature. Then, the fluorescence was measured using the VERITAS microplate luminometer (Turner Biosystems).

### Annexin V apoptosis assay

Apoptosis was measured using the FITC Annexin V apoptosis detection kit II (BD Biosciences, San Diego, CA, USA). Briefly, cells were resuspended in 1× binding buffer. Then, 50 μL of the solution was transferred to a 1.5-mL tube and 10 μL of Annexin V and 10 μL of PI staining solution were added. Subsequently, the cells were mixed by swirling and incubated for 15 min at room temperature in the dark. Finally, the cells were diluted in 200 μL of phosphate-buffered saline (PBS) for analysis by flow cytometry (FACSAria III, BD Biosciences). Data were processed using the FACSDiva software (BD Biosciences).

### ROS generation detection assay

ROS was measured using the 2',7'-dichlorfluorescein-diacetate (DCFH-DA) fluorescence dye (Molecular Probe, Eugene, OR, USA). Briefly, 1 mL of the cell suspension was transferred to a 1.5-mL culture tube and 1 μL of DCFH-DA staining solution was added. Subsequently, cells were gently mixed and incubated for 15 min at 37°C in the dark. Next, cells were washed again and resuspended in 300 μL of cold PBS. Finally, ROS generation was monitored using a flow cytometer (FACSAria III, BD Biosciences), and data were processed with the FACSDiva software (BD Biosciences).

### Mitochondrial membrane potential (JC-1) assay

MMP was assessed by staining the cells with the JC-1 fluorescence dye (Molecular Probe). Briefly, 1 mL of the cell suspension was transferred to a 1.5-mL culture tube and 0.5 μL of JC-1 staining solution was added to cells. Next, the cells were gently mixed and incubated for 5 min in an incubator (37°C) in the dark. Cells were washed again and resuspended in 300 μL of cold PBS. MMP was analyzed using flow cytometry (FACSAria III, BD Biosciences), and data were processed with the FACSDiva software (BD Biosciences).

### Two-photon (TP) fluorescence microscopy

TP fluorescence microscopy images of SHP-Mito-labeled cells were obtained with spectral confocal and multiphoton microscopes (Leica TCS SP8MP) with ×40 oil and ×63 oil objectives, numerical apertures (1.30 and 1.40, respectively). TP fluorescence microscopy images were obtained with a DMI6000B Microscope (Leica) by exciting the probe with a mode-locked titanium-sapphire laser source (Mai Tai HP; Spectra Physics, 80 MHz pulse frequency, 100 fs pulse width) set at a wavelength of 750 nm. To obtain images in the 400–470 nm (blue) and 530–600 (yellow) ranges, internal photomultiplier tubes were used to collect the signals in 8-bit unsigned 1024 × 1024 pixels at a scan speed of 400 Hz. Ratiometric image processing carried out using MetaMorph software.

### Western blot analysis

After treatment, proteins were isolated by resuspending the cells in M-PER mammalian protein extraction reagent (Thermo Scientific, Waltham, MA, USA) supplemented with the Halt protease inhibitor cocktail (Thermo Scientific). Extracts were loaded onto 10% polyacrylamide gels and transferred to Hybond enhanced chemiluminescence (ECL) membranes (GE Healthcare Life Sciences, Little Chalfont, Buckinghamshire, UK). Membranes were blocked with 5% skim milk. The membranes were then incubated with specific primary antibodies, i.e., p-p53 (ser15) (1:1000) and β-actin (1:1000) (Santa Cruz Biotechnology, Santa Cruz, CA, USA) and p53 (Millipore, MA, USA), which were diluted with PBS containing 0.05% Tween 20 (PBST), at 4°C with gentle shaking overnight. After several cycles of washing, the membranes were incubated for 2 h with ImmunoPure goat anti-mouse or anti-rabbit IgG peroxidase-conjugated antibody (Thermo Scientific) diluted in PBST (1:1,000). After washing several times with PBST, proteins were detected using the Pierce ECL western blotting substrate (Thermo Scientific). The blots were exposed to autoradiography films, which were analyzed with the LAS-1000 system (FUJIFILM).

### RNA isolation and qRT-PCR

The RNeasy mini kit (QIAGEN, Valencia, CA, USA) was used to isolate total RNA. Then, 500 ng of total RNA was reverse transcribed to cDNA with a reverse transcription kit (QIAGEN). PCR was performed using the Maxima SYBR Green/ROX qPCR master mix (Thermo Scientific). Thermal cycling was performed with an initial denaturation phase of 10 min at 95°C, followed by 40 cycles of 30 s at 95°C, 30 s at 57°C, and 1 min at 72°C. Amplifications were carried out in the Rotor-Gene Q (QIAGEN). Melting curve analysis was performed to confirm the specificity of the results and reagents. For relative quantification, we used the ΔΔCT method with the Rotor-Gene Q Series Software version 2.2.3. The primers used in this study are *ACMSD*: F: 5′-GGT GCG AGA GAA TTG CTG G-3′, R: 5′-TGC TGG CAA GGT CGT TGT TT-3′; *BIK*: F: 5′-GAC CTG GAC CCT ATG GAG GAC-3′, R: 5- CCT CAG TCT GGT CGT AGA TGA-3′; *ENDOG*: F: 5′-AGC AGG TGG GCA AAT TGA G-3′, R: 5′-CCA GGA TGT TTG GCA CAA AGA G-3′; *HIF1α*: F: 5′-CAC CAC AGG ACA GTA CAG GAT-3′, R: 5′-CGT GCT GAA TAA TAC CAC TCA CA-3′; *HK2*: F: 5′-GAG CCA CCA CTC ACC CTA CT-3′, R: 5′-CCA GGC ATT CGG CAA TGT G-3′; *OPA1*: F: 5′-TGT GAG GTC TGC CAG TCT TTA-3′, R: 5′-TGT CCT TAA TTG GGG TCG TTG-3′; *PDK1*: F: 5′-CTG TGA TAC GGA TCA GAA ACC G-3′, R: 5′-TCC ACC AAA CAA TAA AGA GTG CT-3′; *PRKAG2*: F: 5′-TGC CCG TTA TTG ACC CTA TCA-3′, R: 5′-CAG GCT TTG GCA TAT CAG ACA T-3′; *RRM2B*: F: 5′-AGA GGC TCG CTG TTT CTA TGG-3′, R: 5′-GCA AGG CCC AAT CTG CTT TTT-3′; *β-actin*: F: 5′-ATA GCA CAG CCT GGA TAG CAA CGT AC-3′, R: 5′-CAC CTT CTA CAA TGA GCT GCG TGT G-3′.

### Microarray analysis

Total RNA was extracted using an RNeasy mini kit (QIAGEN), and the concentration in the samples was measured using a Micro UV-Vis fluorescence spectrophotometer (Malcom). Probe labeling and hybridization to Affymetrix GeneAtlas Human Genome U219 chips containing 49,386 probe sets were then carried out. Microarray data were normalized using the robust multi-array average analysis via the Affymetrix Expression Console software (ver. 1.1) (http://www.affymetrix.com/support). When examining the cisplatin-induced differences in the expression profiles, only genes with an average log_2_-(treated/control) (in absolute values) ≥ 1 (which corresponded to a 2-fold change in the expression) at *p* < 0.01 were considered differentially expressed. Moreover, the data have been submitted to Gene Expression Omnibus (GEO) database and the GEO accession number is GSE69644.

### Data processing and statistical analysis for proteome profiling

Liquid chromatography-tandem mass spectrometry (LC-MS/MS) raw data were searched using the SEQUEST algorithm against the ipi.HUMAN.v3.86 database with decoy sequences using the following parameters: 2.0 Da precursor mass tolerance; 1.0 Da product ion mass tolerance; fully tryptic digestion; up to two missed cleavages; variable modifications: oxidation of methionine (+15.99), carbamidomethylation of cysteine (+57.02), and acetylation of the N-terminus (+43.02). PeptideProphet probability was computed using TPP v.4.5 [18] to evaluate the SEQUEST search results. Peptides with a prophet error rate of <0.01 were regarded as identified peptides and quantified using a spectral count-based method previously suggested [19]. In this method, the number of identified spectra assigned to each peptide from each LC-MS/MS run (nine replicates for 0 h and 6 h and seven replicates for 24 h) was counted as peptide-level spectral counts. Then, the peptide-level spectral counts computed from each replicate were summed and divided by the number of replicates to calculate peptide-level average spectral counts. With the peptide-level average spectral counts measured from 0 h, 6 h, and 24 h samples, the R_SC_s, the log_2_ ratio of abundance between two samples [20], were calculated for two comparisons, i.e., 6 h/0 h and 24 h/0 h. To evaluate the statistical significance of the R_SC_ value for each peptide, empirical distributions of the R_SC_ values were generated by 1000 random permutation experiments. Peptides whose absolute R_SC_ values are above the 99^th^ percentile value of the R_SC_s in the empirical distributions (1.64 for 6 h and 1.82 for 24 h) were regarded as differentially expressed peptides. Proteins that have at least one differentially expressed peptide (DEP) were considered as differentially expressed proteins.

### LC-MS data processing and statistical analysis

Data were normalized by dividing each signal by the sum of the signals for all metabolites in the same run. The Student’s *t*-test was performed for the metabolite profiles from rat urine and serum. Metabolites with a *p*-value < 0.05 were regarded as differentially expressed metabolites. In case of metabolite profiling from HK-2 cells and media, metabolites whose fold increase was >95% compared to that of the change in the empirical distribution were selected as differentially expressed metabolites.

### Functional enrichment analysis

Analysis of the 5,692 DEGs and 487 DEPs was performed using the Database for Annotation, Visualization, and Integrated Discovery (DAVID) software. The results were analyzed with the enrichment map plug-in for Cytoscape, with which functional enrichment can be visualized and compared [21–23].

### Animals and husbandry

Male Sprague-Dawley rats (weighing approximately 180 g, 6 weeks old) were used. They were kept in a controlled environment (at 22°C ± 2°C with 50–60% relative humidity) during a 12-h light-dark cycle. Rats were allowed to acclimate for 1 week before the experiments and randomly allocated into control and drug treatment groups with six rats per group. Animal experiments were conducted in accordance with the Guide for Animal Experiments published by the Korea Academy of Medical Sciences. The protocol was approved by the Pusan National University–Institutional Animal Care and Use Committee (PNU-IACUC) (Permit Number PNU-2011-00211). All efforts were made to reduce their suffering.

### *In vivo* experimental design

Rats were administered intraperitoneally (i.p.) a single dose of cisplatin (10 mg/kg, dissolved in 0.9% saline) on day 0. It has been previously shown that this dose results in proximal tubule cell necrosis or apoptosis, and severe damage in kidney function within 3 days after the cisplatin injection. The vehicle control group was injected with 0.9% saline in the same manner. Urine samples of cisplatin-treated animals were collected at 0, 1, and 3 days. Each animal was kept in a rat metabolic cage overnight and 24-h urine samples were collected in the morning. Animals were monitored daily by the disease activity index that includes loss of body weight, serum levels of blood urea nitrogen (BUN) and creatinine, and hair loss as described previously [24–26]. Animals were euthanized under anesthesia by cervical dislocation when they lost more than 20% of starting bodyweight.

### ¹H-NMR spectroscopy and data processing

Analysis of the urine samples was carried out on a Varian system (Varian Medical Systems, Palo Alto, CA, USA) with a working frequency of 600.167 MHz at a temperature of 299.1 K. Deuterium oxide and 3-(trimethylsilyl propionic-2,2,3,3-d4 acid) were used as field-lock frequency and internal chemical shift reference, respectively. A single-pulse spectrum was obtained using a Carr-Purcell-Meiboom-Gill pulse sequence to suppress the water signal. For each ¹H-NMR spectrum, 128 scans were collected into 32K data points, which resulted in an acquisition time of 12 min. All data were automatically apodized with an exponential function using a line broadening of 0.2 Hz prior to Fourier transformation and calibrated to 3-(trimethylsilyl propionic-2,2,3,3-d4 acid) at δ = 0.00 ppm. Spectral assignment was performed using the Chenomx NMR Suite 7.1 software (Chenomx, Edmonton, AB, Canada) and compared to data published in the literature. After processing, data were reduced into 920 spectral integral regions equating to the chemical shift range of δ (0.2–10 ppm) using the Chenomx NMR Suite.

### NMR multivariate data analysis

All binned NMR data were analyzed by principal component analysis (PCA) using the SIMCA-P+ 12.0 software (Umetrics, Tvistevägen, Umeå, Sweden). PCA is one of the pattern recognition methods and useful to detect differences in each spectrum. To clarify the differences among samples, orthogonal-projection to latent structure-discriminant analysis was applied.

### Statistical analysis for metabolite profiling

Data were normalized by dividing each signal by the sum of the signals measured from the same run. Metabolites with a Student’s *t*-test *p*-value < 0.05 were regarded as differentially expressed metabolites.

## Results

### Cisplatin induced dose-dependent toxicity in HK-2 cells

**Fig 1:**
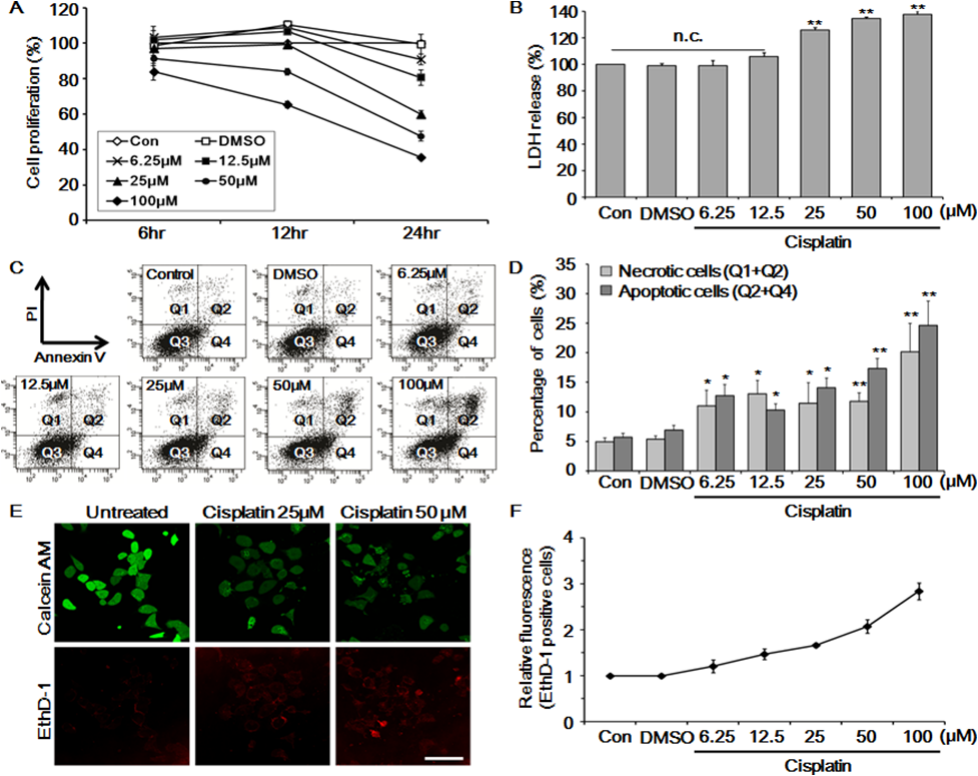
Evaluation of cellular toxicity and viability in cisplatin-treated HK-2 cells. HK-2 cells were treated with increasing concentrations of cisplatin (6.25–100 μM). (A) HK-2 cell proliferation was assessed by MTS assay after 6, 12, and 24 h of cisplatin treatment. (B) Cells were treated with the indicated concentrations of cisplatin for 24 h and cell death was measured. (C and D) HK-2 cells were washed twice with cold phosphate-buffered saline (PBS), stained with Annexin V/propidium iodide (PI), and cell death was determined by flow cytometry. (E and F) Cisplatin-induced cell viability and cytotoxicity assay using calcein AM and ethidium homodimer (EthD-1) fluorescence staining with confocal microscopy (E) and flow cytometry (F). Magnification of the images was ×40 and scale bars represent 75 μm. The mean ± S.D. (standard deviation) of three independent experiments are presented; *p < 0.05, **p < 0.01, by paired *t*-test.

To evaluate its toxicity, HK-2 cells were treated with varying concentrations of cisplatin (6.25, 12.5, 25, 50, or 100 μM) for 6, 12, and 24 h. We found that cell proliferation was decreased in a dose- and time-dependent manner. Cell viability decreased to 52.5% ± 2% in the group treated with 50 μM for 24 h (Fig 1A) implying a half-maximal inhibitory concentration as of 50 μM. Next, cell damage was quantified in cells treated with cisplatin for 24 h using the lactate dehydrogenase (LDH) assay (Fig 1B). The result showed that 34.53% and 37.64% of cells were damaged after treatment with 50 and 100 μM of cisplatin, respectively. Next, cell viability was evaluated using propidium iodide (PI) and annexin V double-stain method (Fig 1C and 1D). The results showed that 17.30% and 24.63% of cells were apoptotic and 11.77% and 20.13% were necrotic at 50 μM and 100 μM cisplatin, respectively.

Similar results were observed when the fluorescence intensity of cells stained with the dyes calcein AM and ethidium homodimer (EthD-1) was measured (Fig 1E). Calcein AM is incorporated into live cells where it produces green fluorescence, whereas EthD-1 binds to the DNA of dead cells and produces red fluorescence [27]. From the fluorescence intensity, it was apparent that the red fluorescence intensity increased proportionally to the cisplatin concentration. Using fluorescence-activated cell sorting (FACS), quantitative data were obtained for the increase in EthD-1 fluorescence intensity (Fig 1F) that corresponded to a 2.1- and 2.8-fold increase at 50 μM and 100 μM of cisplatin, respectively, compared to that of control cells.

These results demonstrated that cisplatin causes cell death in HK-2 cells. Furthermore, considering these observations, 50 μM cisplatin (half-maximal inhibitory concentration) was used in subsequent experiments.

### Cellular gene ontology analysis showed significant expression profiles and functional interactions

To examine transcriptome perturbations, mRNA levels were determined using the Affymetrix GeneAtlas system and U219 array. Data were analyzed using the Affymetrix Expression Console software and 2-fold transcriptional changes were considered significant at *p <* 0.01. Cellular protein expression was analyzed using the liquid chromatography-tandem mass spectrometry (LC-MS/MS) method. The number of identified spectra assigned to each peptide for each LC-MS/MS run was counted. Peptides with an absolute value of the relative spectral count (R_SC_) larger than the cutoff value (99^th^ percentile of R_SC_ values obtained from 1000 random permutation experiments; see Materials and Methods section) were regarded as differentially expressed peptides (DEPs). As a result, 487 proteins with at least one DEP from 6 h/0 h or 24 h/0 h comparisons were selected as DEPs. Therefore, gene ontology functions for those 5,692 differentially expressed genes (DEGs) and 487 DEPs were analyzed using the DAVID software. DAVID provides online functional annotation tools for the analysis of sets of genes, especially omics data, including microarray and high-throughput proteomic data [28,29], which were visualized using Cytoscape [21,22].

**Fig 2:**
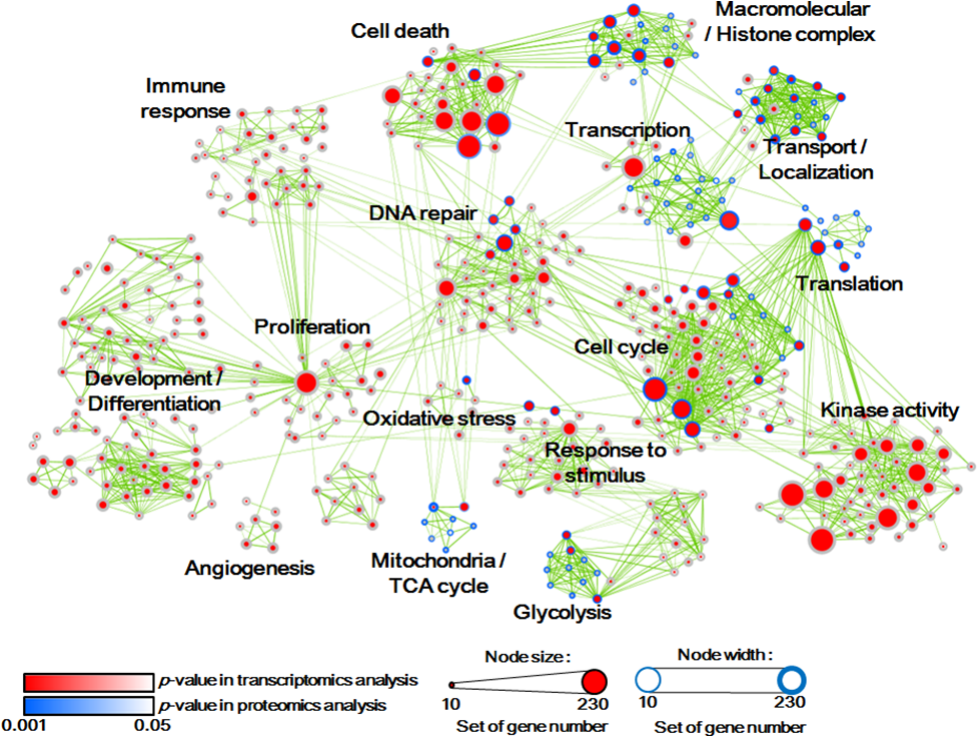
Functional analysis to visualize transcriptomic and proteomic data. Significantly altered genes in HK-2 cells following cisplatin treatment as detected by microarray analysis (transcriptomic) and liquid chromatography coupled with tandem mass spectrometry (LC-MS/MS) (proteomic). Functional analysis of data was performed using the Database for Annotation, Visualization, and Integrated Discovery (DAVID). Results were visualized using the enrichment map plug-in for Cytoscape. Each circular node is a gene set with a diameter proportional to the number of genes. The inner node color represents the *p*-value of the enrichment analysis in the transcriptome, whereas the outer node color represents the *p*-value of the enrichment analysis in the proteome. The edge width is proportional to the similarity of gene sets between linked nodes. Details of the network can be examined using the scalable PDF in S1 Fig.

The DAVID network derived from the effects of cisplatin in HK-2 cells provided an overview of the functional analysis (Fig 2). The complete functional network after identification of all labeled nodes has been reported (S1 Fig). Each circular node corresponds to a gene set and the circle size indicates the number of genes in it. The color of the inner and outer nodes represents the *p*-value of the enrichment analysis of transcriptomic data and proteomic data, respectively. Each edge of a gene set represents gene overlap among gene sets and thickness of the edges indicates similarity of genes among linked nodes [23].

Cisplatin causes changes in cell physiology, in particular, cell viability, cell cycle, DNA repair, mitochondria, glycolysis, and transport function that were evident in both the transcriptomic and proteomic analysis. We focused on functional anomalies in mitochondrial metabolism and glycolysis and constructed a detailed functional map using STITCH and Cytoscape. Using the STITCH database, protein-protein interactions in each homologue gene group have been identified [30–32] and displayed through Cytoscape (S2 Fig). Each node indicates a gene group, where the square node represents protein expression and the circular node represents mRNA expression. The node color represents fold changes in genes expression, i.e., red indicates up-regulation and green indicates down-regulation. From these results, we identified proteins and mRNAs with significant expression profiles and their functional interaction with cisplatin in HK-2 cells.

### Cisplatin induced metabolic abnormalities in rat urine samples

**Fig 3:**
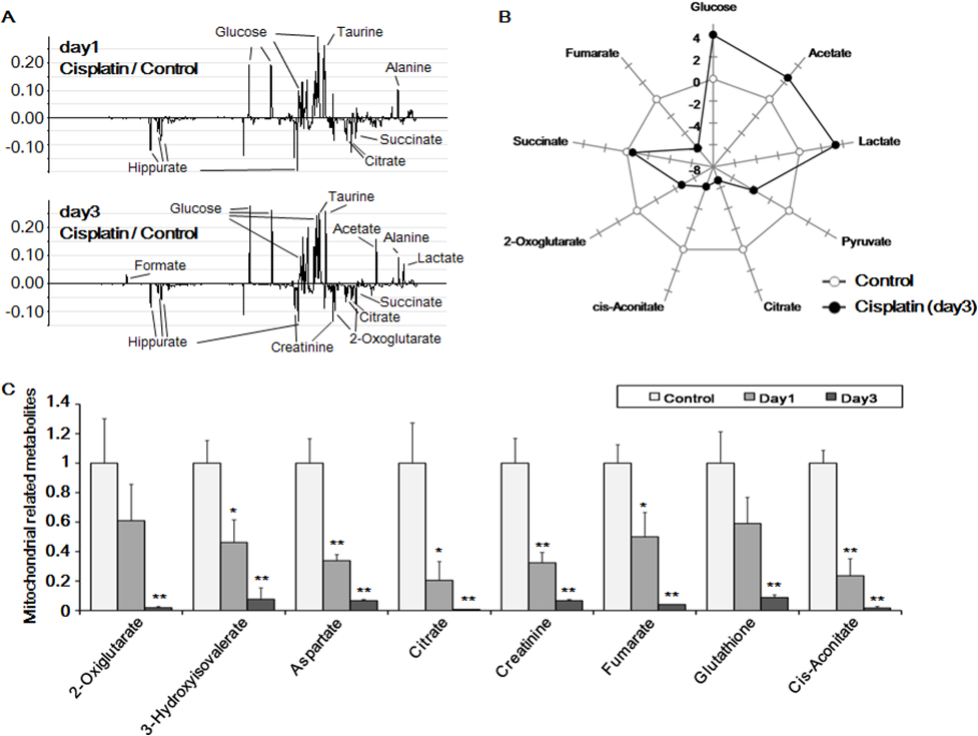
Metabolite profiling of cisplatin-treated rat urine samples. (A) Proton nuclear magnetic resonance (^1^H-NMR) spectra of day 1 (upper) and day 3 (lower) after cisplatin injection compared with those of controls. (B) Alterations of glycolysis- and tricarboxylic acid (TCA) cycle-related metabolites compared with control samples. (C) Effect of cisplatin on the level of mitochondria-related metabolites in urine samples. The mean ± S.D. of three independent experiments are presented; *p < 0.05, **p < 0.01, by paired *t*-test.

To correlate the in vitro results to in vivo effects, rats were administered cisplatin. Urine was collected at days 0, 1, and 3 post-injection and subjected to proton nuclear magnetic resonance (^1^H-NMR) analysis. Fig 3A shows rat urine ^1^H-NMR spectra from day 1 (upper) and day 3 (lower) compared to control spectra. Only metabolites related to glycolysis and the TCA cycle were analyzed (Fig 3B).

Interestingly, urine glucose levels were increased at day 3 after cisplatin administration, whereas pyruvate levels were decreased compared to control. All TCA-related metabolites were decreased in urine following cisplatin treatment (Fig 3C). The decrease in TCA metabolites could be caused by inhibition of glycolysis or damage to mitochondria. Inhibition of glycolysis leads to decreased production of pyruvate, a starting compound of TCA cycle. Moreover, the TCA cycle occurs in the mitochondrial matrix; therefore, any damage to mitochondria would also inhibit the TCA cycle.

Collectively, these results established that cisplatin blocked the glucose metabolism, which is in agreement with the results of transcriptomic and proteomic analyses in cisplatin-treated HK-2 cells.

### Cisplatin treatment disrupted the mitochondrial membrane potential

ROS reduced cell viability, and cisplatin triggers ROS generation resulting in cell death [33–35]. It was confirmed that ROS level increased in HK-2 cells to 22.54% and 58.93% after cisplatin treatment for 12 and 24 h, respectively (S3A Fig), supporting earlier observations.

**Fig 4:**
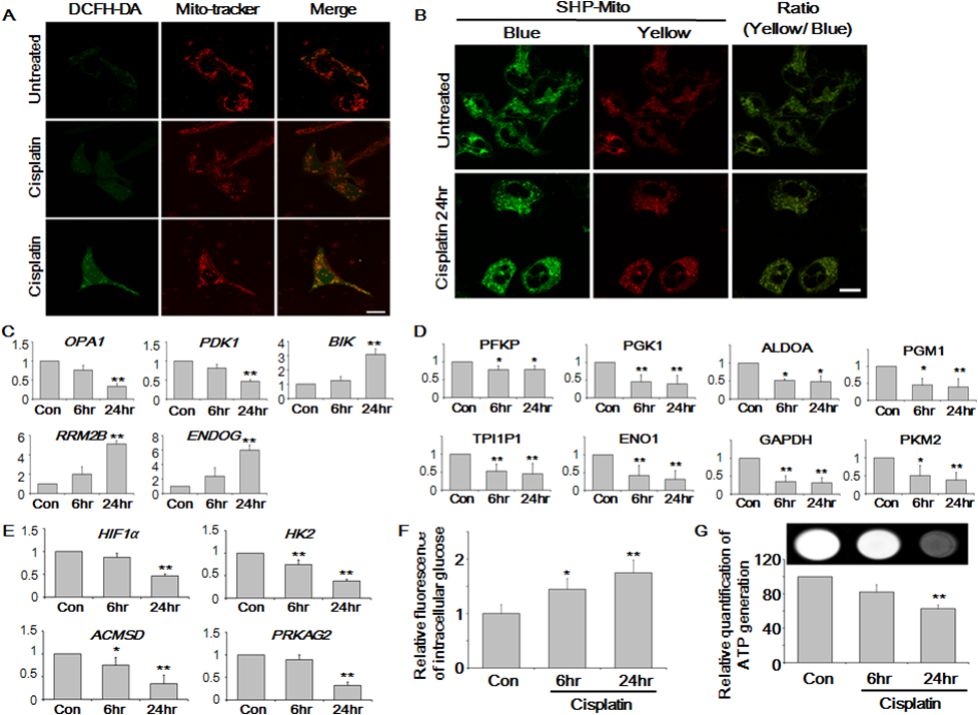
Cisplatin induced ATP depletion via mitochondrial damage and inhibition of glycolysis. (A) Intracellular localization of reactive oxygen species (ROS) by confocal microscopy. Visualization of mitochondria by Mito-tracker Red CMX (red, middle) demonstrated co-localization with mitochondrial ROS (mROS) as detected by the 2′,7′-dichlorofluorescein diacetate (DCFH-DA) (green, left) fluorescence dye. Magnification of the images was ×100 and scale bars represent 30 μm. (B) Quantification of mROS by the two-photon (TP) probe for the ratiometric detection of mitochondrial H_2_O_2_. The reaction between the SHP-Mito probe and H_2_O_2_ resulted in a change in the fluorescence from blue to yellow. Pseudo-colored green areas indicated mitochondria (blue fluorescence, left), and pseudo-colored red areas indicate mitochondrial H_2_O_2_ (yellow fluorescence, middle). Magnification of the images was ×63 and scale bars represent 26 μm. (C) mRNA expression levels of mitochondria-related genes (*OPA1*, *PDK1*, *BIK*, *RRM2B*, and *ENDOG*) determined after cisplatin treatment by quantitative reverse transcriptase-polymerase chain reaction (qRT-PCR). (D) Expression levels of glycolysis-related enzymes of cisplatin-treated cells using liquid chromatography coupled with tandem mass spectrometry (LC-MS/MS) analysis. (E) mRNA expression levels of glycolysis-related genes (*HIF1a*, *HK2*, *ACMSD*, and *PRKAG2*) determined after cisplatin treatment using qRT-PCR. (F) Quantification of intracellular glucose levels after cisplatin treatment. (G) Intracellular ATP levels were determined using LAS 1000 and a microplate reader. The mean ± S.D. of three independent experiments are presented; *p < 0.05, **p < 0.01, by paired *t* test.

Cisplatin rapidly accumulates in mitochondria and induces oxidative stress that may damage mitochondrial DNA [13,36–38]. Therefore, it was proposed that ROS are generated from mitochondria due to accumulation of cisplatin. To confirm this, the location of ROS generation was investigated in cisplatin-treated cells co-stained with Mito-tracker-Red CMX (Molecular Probe, NY, USA) and the 2′,7′-dichlorofluorescein diacetate (DCFH-DA) fluorescence dye (Fig 4A and S5A Fig). Co-staining revealed that the location of intracellular ROS generation coincided with that of mitochondria. The overlap of Mito-tracker-Red-stained areas with DCFH-DA-positive areas was >75% in cells after 24 h treatment. These co-localization results demonstrated that cisplatin-induced mitochondrial ROS (mROS) generation is associated with mitochondrial deterioration in HK-2 cells.

Next, the amount of mROS was determined using a two-photon (TP) probe for the ratiometric detection of mitochondrial H_2_O_2_. This TP probe was derived from 6-(benzo[*d*]thiazol-2pryl)-2-(*N*,*N*-dimethylamino) naphthalene as the reporter, a boronate-based carbamate leaving group as the H_2_O_2_ response site, and triphenylphosphonium salt as mitochondrial targeting site. The reaction between the SHP-Mito probe and H_2_O_2_ would turn blue fluorescence into yellow fluorescence [39]. Accordingly, the yellow/blue fluorescence ratio of mitochondrial H_2_O_2_ increased up to 0.92 in cells treated with cisplatin for 24 h compared to untreated cells (0.63) (Fig 4B and S5A Fig).

Morphologically, mitochondria from cisplatin-treated cells appeared disintegrated with SHP-Mito probe when observed at digital high magnification (S4B Fig). Mitochondrial fragmentation has been shown to occur during cell injury and apoptosis and might be the result of the activation of fission [40]. Next, the MMP was evaluated using the JC-1 fluorescence dye, whose ratio of green-to-red fluorescence depends only on the membrane potential [41,42]. It was observed that the fluorescence changed from red to green following cisplatin treatment in a time-dependent manner (S3B Fig).

Furthermore, to confirm mitochondrial abnormalities, the expression level of genes related to mitochondrial function—optic atrophy 1 (*OPA1*), pyruvate dehydrogenase kinase 1 (*PDK1*), BCL2-interacting killer (*BIK*; apoptosis-inducing), ribonucleotide reductase M2 B (*RRM2B*), and endonuclease-G (*ENDOG*)—were evaluated by quantitative reverse transcription polymerase chain reaction (qRT-PCR) on the basis of transcriptomic data as shown in S2 Fig (Fig 4C). *Opa1* is related to mitochondrial fission and fusion [43,44], whereas *PDK1* is critical in conserving mitochondrial functions [45]; both genes were down-regulated with time, which is in agreement with previous results (Fig 4C). *ENDOG*, *RRM2B*, and *BIK* are markers of mitochondrial DNA damage and oxidative stress that are vital in a multitude of physiological aspects [46–50]; all three genes were up-regulated in the presence of cisplatin.

Altogether, these results suggested that cisplatin causes mitochondrial dysfunction, fragmentation, and collapse of the membrane potential, via increased mROS generation.

### Cisplatin-mediated inhibition of glycolysis led to increased cellular glucose levels

Using LC-MS/MS, the expression level of enzymes involved in glycolysis, phosphofructokinase platelet, phosphoglycerate kinase 1, aldolase A, phosphoglucomutase 1, triosephosphate isomerase 1 pseudogene 1, enolase 1, glyceraldehyde-3-phosphate dehydrogenase, and pyruvate kinase muscle 2, were determined after 6 and 24 h of cisplatin treatment (Fig 4D) and found to be down-regulated compared to the control. A reduction in the levels of these enzymes would inhibit glycolysis.

Next, the expression levels of glycolysis pathway-related genes (*HIF1α*; hexokinase 2 [*HK2*]; protein kinase, AMP-activated, gamma 2 non-catalytic subunit [*PRKAG2*]; and aminocarboxymuconate semialdehyde decarboxylase [*ACMSD*]) were evaluated (Fig 4E), because transcriptomic data indicated significant changes in their expression levels (S2 Fig). *HIF1α* induces glycolysis enzymes [51], *HK2* is the first enzyme of glycolysis [44], and *PRKAG2* modulates glycolysis [52,53]. *ACMSD* acts as a regulatory link between glycolysis and nicotinamide adenine dinucleotide synthesis [54]. All of these enzymes are necessary for the proper execution of glycolysis, but their expression levels decreased over time. This, in turn, resulted in abnormal glycolysis and increased intracellular glucose levels (up to 75%) (Fig 4F), which also confirmed the results of the omics data analysis.

### Cisplatin lowered ATP levels via mitochondrial dysfunction and glycolysis inhibition

Mitochondria are the vital organelles in cells to produce energy and have a central role in cell proliferation and death signaling [40]. With the collapse of MMP (Δψm), mitochondrial deterioration [55] or glycolysis disturbance [56], ATP depletion occurs.

To evaluate the ATP level in response to cisplatin treatment, the intracellular ATP level was quantified using a luminescence assay (Fig 4G). Results showed that the ATP level decreased in a time-dependent manner; particularly, in cells treated with cisplatin for 24 h, the ATP level was reduced by 37.25% compared to untreated cells.

### Cisplatin inhibited glycolysis via p53 activation, irrespective of mitochondrial damage

Several studies have reported that cisplatin activates p53 [57,58]. Activated p53 inhibits glycolysis via inhibition of HIF1α or induction of TP53-induced *glycolysis* and apoptosis regulator [8–11].

**Fig 5:**
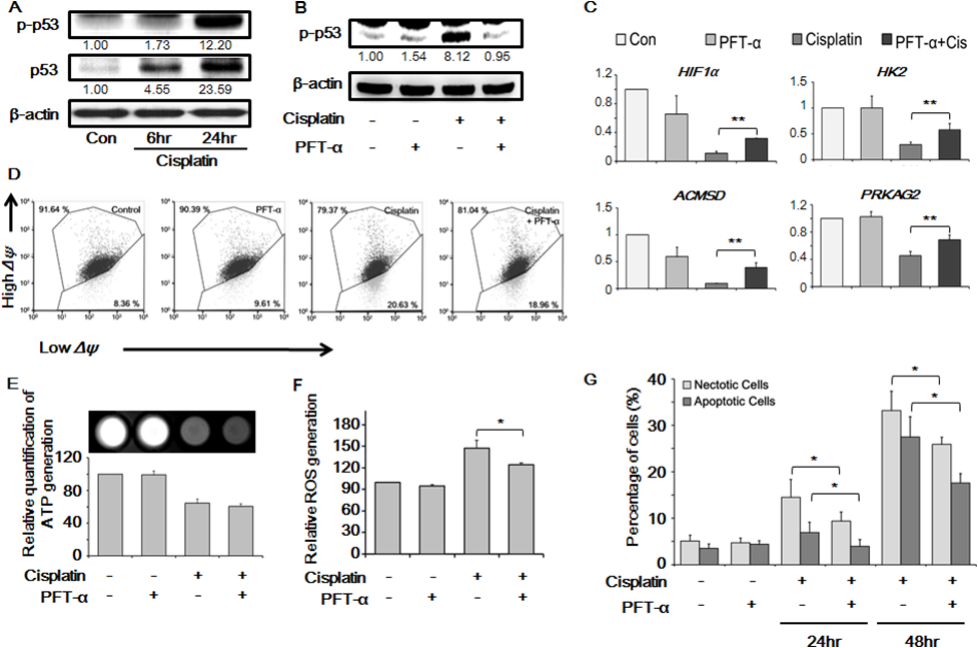
Cisplatin inhibited glycolysis via p53 activation. (A) Western blot analysis indicated increased levels of p53 and phospho-p53 after 6 and 24 h of cisplatin treatment. The gels have been run under the same experimental conditions. (B) Expression of p53 in cells treated with pifithrin-α (PFT-α) (20 μM) was analyzed by western blot. The gels have been run under the same experimental conditions. (C) mRNA expression levels of glycolysis-related genes (*HIF1a*, *HK2*, *ACMSD*, and *PRKAG2*) determined in cisplatin-treated and cisplatin/PFT-α (20 μM) co-treated cells by quantitative reverse transcriptase-polymerase chain reaction (qRT-PCR). (D) Δψ_m_ depolarization monitored by FACS analysis with the JC-1 dye where a fluorescence shift from red to green indicated the collapse of the mitochondrial membrane potential (MMP). The number of cells with low MMP was counted. (E) The intracellular ATP level was measured using LAS 1000 and a microplate reader. (F) Reduced levels of intracellular reactive oxygen species (ROS) were observed in HK-2 cells with 2′,7′-dichlorofluorescein diacetate (DCFH-DA) following treatment with PFT-α (20 μM). (G) Treatment with a p53 inhibitor prevented cisplatin-induced cell death at 48 h in HK-2 cells when analyzed by Annexin V/propidium iodide (PI) staining. The percentage of apoptotic cells was determined by flow cytometry. The mean ± S.D. of three independent experiments are presented; *p < 0.05, **p < 0.01, by paired *t*-test.

Cisplatin induced total p53 and phosphorylated p53 (Fig 5A). When p53 was inhibited by 20 μM pifithrin-α (PFT-α) (Sigma-Aldrich) in cisplatin-treated cells and the expression levels of glycolysis-related genes were determined by qRT-PCR (Fig 5B), it was found that the expression of genes suppressed by cisplatin induced p53 were restored. However, MMP and cellular ATP levels were not affected (Fig 5D and 5E), while a decline in intracellular ROS levels was observed by p53 inhibition (Fig 5F). Next, cell death was evaluated using PI and annexin V double-staining (Fig 5G). Interestingly, in line to these observations, in PFT-α-treated cells, the rate of cell death was significantly different for up to 24 h, and similar trend was observed after 48 h.

Finally, the intracellular glucose and lactate levels have been evaluated as p53 is the inhibitor of glycolysis. When p53 was inhibited by PFT-α, intracellular glucose and lactate levels have been significantly decreased, implying the restoration of glycolysis (S4A and S4B Fig).

### Altered cellular events were restored by inhibition of ROS generation

As reported earlier, cisplatin-induced cytotoxicity is closely related to increased mROS generation [4,5]. Therefore, we investigated whether cisplatin-induced mROS generation was the cause of the cellular damage phenomenon, including mitochondrial damage and exacerbation of glycolysis.

**Fig 6:**
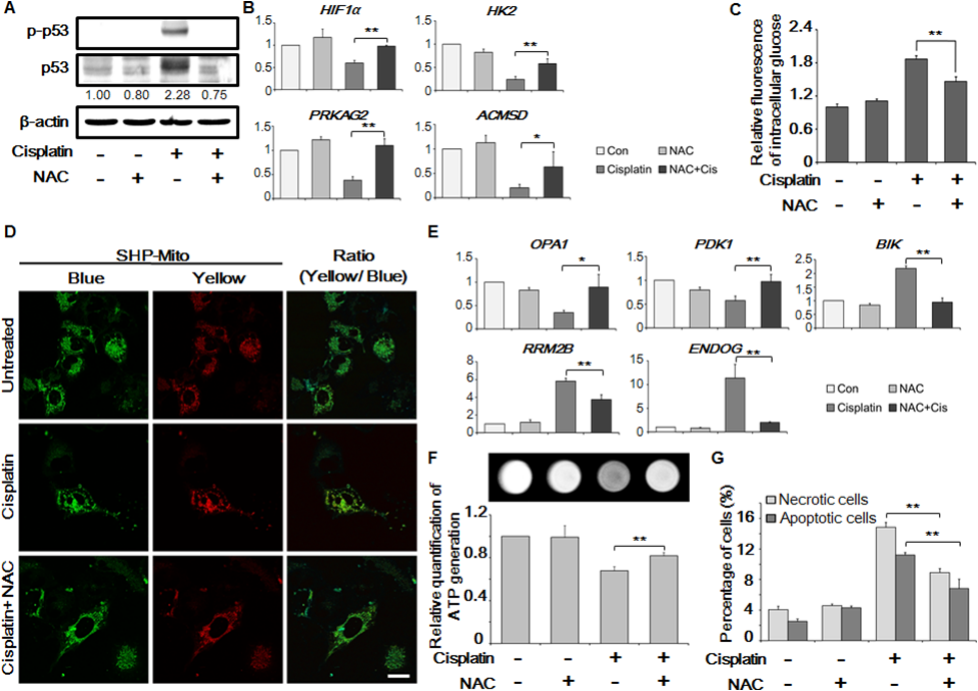
Restoration of altered cellular events by inhibition of mitochondrial reactive oxygen species (mROS) generation. (A) Cisplatin-mediated expression and activation of p53 was evaluated and compared with cisplatin and *N*-acetylcysteine (NAC) (10 μM) co-treated samples by western blot. The gels have been run under the same experimental conditions. (B) mRNA expression levels of glycolysis-related genes (*HIF1a*, *HK2*, *ACMSD*, and *PRKAG2*) were determined in cisplatin and NAC co-treated cells by quantitative reverse transcriptase-polymerase chain reaction (qRT-PCR). (C) NAC reversed the cisplatin-induced increase in the intracellular glucose concentration. (D) The amount of mROS was measured using a TP probe for the ratiometric detection of mitochondrial H_2_O_2_. The reaction between the SHP-Mito probe and H_2_O_2_ resulted in a change in the fluorescence from blue to yellow. Pseudo-colored green areas indicate mitochondria (blue fluorescence, left) and pseudo-colored red areas indicate mitochondrial H_2_O_2_ (yellow fluorescence, middle). NAC decreased mitochondrial H_2_O_2_ generation. Magnification of the images was ×63 and scale bars represent 30 μm. (E) mRNA expression levels of mitochondria-related genes (*OPA1*, *PDK1*, *BIK*, *RRM2B*, and *ENDOG*) were determined in cisplatin and NAC co-treated cells by qRT-PCR. (F) The intracellular ATP level was restored following NAC treatment, as quantified by LAS 1000 and a microplate reader. (G) NAC prevented cisplatin-induced cell death as evaluated by Annexin V/PI staining, and the ratio of apoptotic and necrotic cells was determined using flow cytometry. The mean ± S.D. of three independent experiments are presented; *p < 0.05, **p < 0.01, by paired *t*-test.

*N*-acetylcysteine (NAC) is a powerful antioxidant, a precursor of glutathione [3] that can scavenge ROS to suppress its activity [2]. Therefore, cisplatin-induced ROS generation was suppressed using NAC to elucidate the cisplatin-induced abnormalities that have been observed previously in HK-2 cells. First, the effect of NAC on cisplatin-induced ROS generation was studied using the DCFH-DA dye (S6A Fig). NAC significantly reduced cisplatin-dependent intracellular ROS generation. Similarly, inhibition of ROS generation reduced expression and phosphorylation of p53 (Fig 6A).

Next, the expression levels of glycolysis-related genes were analyzed in cisplatin and NAC co-treated cells by qRT-PCR (Fig 6B). Compared to treatment with cisplatin alone, *HIF1α*, *HK2*, *ACMSD*, and *PRKAG2* were significantly up-regulated in cells co-treated with NAC. Moreover, the cisplatin-induced increase in the intracellular glucose level was also significantly reduced by co-treatment with NAC (186.8% to 131.3%) (Fig 6C).

Furthermore, the effect of suppressed mROS generation on the mitochondrial status was evaluated using SHP-Mito TP probe (Fig 6D). We found that the yellow/blue fluorescence signal from mitochondrial H_2_O_2_ decreased in NAC and cisplatin co-treated cells (0.79) compared with cells treated with cisplatin alone (0.98) (S5C Fig). Mitochondrial fragmentation caused by cisplatin was also ameliorated by NAC treatment (S5D Fig).

The expression level of the mitochondrial fission suppression gene *OPA1*, and *PDK1* were significantly restored in the presence of NAC (Fig 6E). The apoptosis signal inducible genes *BIK*, *RRM2B*, and *ENDOG*, which are present in mitochondria, were also significantly down-regulated to their normal levels. These results demonstrated that NAC reversed cisplatin-induced cellular damages.

Finally, intracellular ATP levels were investigated. By inhibiting mROS generation, mitochondrial dysfunction was improved and glycolysis was restored (Fig 6B–6E). This, in turn, led to restoration of intracellular ATP levels, which were increased in cells co-treated with NAC and cisplatin compared with cells treated with cisplatin alone (Fig 6F). The percentages of annexin V- and PI-positive cells were 12.2% and 14.83%, respectively, in cells treated with cisplatin alone; however, in NAC pre-treated samples, the respective values were 6.17% and 8.9% (Fig 6G). Cell viability (S6B Fig) (42.27% to 70.36%) and morphology (S6C Fig) were significantly improved and toxicity was reduced in cells co-treated with NAC.

## Discussion

In recent years, several studies reported that cisplatin influenced different pathways, including cell cycle arrest, apoptosis, proliferation, DNA repair, as well as the TCA cycle and glycolysis [14,15,59]. However, it remained elusive how cisplatin affects metabolic pathways. Here, using transcriptomic and proteomic data, the detailed mechanism has been unveiled, and a map depicting the interactions among various genes has been drawn that clearly indicates the down-regulation of different enzymes.

To analyze the cytotoxicity, different concentrations of cisplatin were applied to HK-2 cells, which reduced cell viability and increased cell death in a dose-dependent manner (Fig 1). Next, genome-wide analyses using microarray and LC-MS/MS revealed perturbations of numerous pathways in cisplatin-treated cells (Fig 2). Many reports correlated cell cycle- and DNA repair-related pathways to cisplatin toxicity; therefore, we analyzed metabolic enzymes in cisplatin-induced cell death. Various glycolysis- and TCA cycle-related enzymes were down-regulated, as shown in microarray and LC-MS/MS analyses (S2 Fig).

In vivo experiments involving rats confirmed the metabolic alterations, i.e., the level of various metabolic intermediates were significantly decreased in cisplatin-treated rats (Fig 3B and 3C). This could be attributed to either damage to mitochondria where the TCA cycle occurs [60] or disruption of glycolysis. Glucose and lactate levels were higher than normal; thus, it can be postulated that glucose consumption is inhibited and glycolysis is deviated from pyruvate to lactate, respectively (S4A and S4B Fig). Here, it was also considered that the glucose uptake might be impaired. Nevertheless, microarray and MS data revealed that the glucose transporter was expressed normally (data not shown); furthermore, glucose levels in the cytoplasm were higher, ruling out impaired glucose transport.

Cisplatin down-regulated the genes involved in glycolysis (Fig 4D and 4E), which resulted in glucose accumulation in HK-2 cells, causing ATP shortage (Fig 4F and 4G), and was excreted via the urine (Fig 3B). Furthermore, mitochondrial damage by cisplatin was evidenced through omics data (Figs 2 and 3) and mitochondrial staining experiments. Microscopic analysis revealed enhanced ROS generation that was largely co-localized with mitochondria, causing fragmentation, MMP disruption, and down-regulation of mitochondrial stability markers (Fig 4 and S3B Fig). Hence, it was evident from these results that cisplatin induced inhibition of glycolysis, TCA cycle dysfunction, and mitochondrial damage.

Several studies indicated the essential role of p53 in cisplatin-induced cytotoxicity [61,62]. In addition, it was noted that p53 was also up-regulated and phosphorylated upon cisplatin treatment (Fig 5A), which resulted in the inhibition of the expression of glycolysis-related genes; this effect was significantly reversed when p53 inhibitor (PFT-α) was applied (Fig 5C). PFT-α treatment also reduced the intracellular glucose and lactate levels, implying the p53 role in altered metabolic route (S4A and S4B Fig). However, MMP and ATP depletions were unaffected by PFT-α treatment (Fig 5D and 5E). This might be correlated to dispensable role of p53 in aerobic respiration in cisplatin treatment, which is the principal source of ATP generation. Also, PFT-α could not restore mitochondrial pathways, which strengthens this concept and provides a plausible explanation of unaffected ATP level. Moreover, the results indicated that p53 is downstream of mROS, because PFT-α application, only reversed the p53-dependent pathways, whereas other pathways remained unaffected.

A previous study showed that, soon after its administration, cisplatin accumulated in mitochondria where it stimulated mROS generation that caused mitochondrial damage [63] and induced other cytotoxic effects [4,5]. To confirm this, NAC was applied [64,65], which scavenged mROS and significantly improved the expression levels of mitochondrial markers in treated cells, reduced fragmentation of mitochondria, significantly restored ATP levels, and reduced cell death. Furthermore, the p53-induced decrease in the expression of glycolysis-related genes and intracellular glucose levels were also recovered by treatment with NAC. These observations indicated that mROS generation is an essential mediator of mitochondrial and metabolic dysfunctions induced by cisplatin, which is in line to previous studies where the quenching of mROS or the use of antioxidants leads to the amelioration of cisplatin induced nephrotoxicity [63,66].

Intriguingly, there might a link between mROS and p53, because mROS induced p53 expression when cells were treated with cisplatin (Fig 6A). In fact, when a p53 inhibitor was applied, low levels of mROS were generated (Fig 5F), suggesting a positive-feedback relation between mROS and p53. Interestingly, a cell must undergo apoptosis when it becomes abnormal rendered a positive feedback relation between mROS and p53 possible. In our study, cell death was significantly reversed by inhibiting p53 for 24 h and for 48 h time points.

The mitochondrial inner membrane is vital for ATP generation via the electron-transport chain (ETC). When cells were treated with cisplatin, the membrane potential decreased (S3B Fig), which led to the collapse of energy generation and ATP depletion, sensitizing cells to apoptosis. Moreover, it is unlikely to rule out the possibility of cytochrome C-mediated cell death; hence, MMP and apoptosis are interlinked.

In cells, the main energy is provided in the form of ATP, which is primarily produced by TCA/ETC, and in small quantities, by glycolysis. The ATP level has a central role in cell proliferation and death signaling [40,55,56]. Cisplatin treatment disrupts central catabolic pathways; consequently, intracellular ATP levels decreased that when this condition continues for a longer period, leading to cell death (Fig 4G). Moreover, fatty acid and protein metabolisms were not studied because of their association to the glucose metabolism, therefore, impaired glucose metabolism would certainly affect these pathways.

Cisplatin induced DNA damage is well appreciated in scientific community that triggers p53 activation, which either rescues cells or leads to apoptosis. Alongside this perspective, another view is that ROS is produced endogenously or in response to treatment, that also triggers p53 [67], but the link describing cisplatin treatment and its metabolic disruption was not established. In addition, the interaction of p53 and the ROS originated from mitochondria is crucial to mediate the various cellular effects. ROS is a stress signal that also triggers p53, while the p53 orchestrates the cell cycle and cell death, and metabolic pathways [68–71]. This coordinated interplay of cellular and mitochondrial ROS and p53 can tweak the cellular behavior towards various stress conditions as having been reported by previous studies [69,72]. Moreover, these results demonstrated that mROS is involved in stress-induced signaling upstream of p53 activation, implying mROS role as the second messenger between mitochondrial ETC and stress induced p53 activation.

Recent reports signified that cisplatin accumulated in mitochondria immediately after administration [13,63], so we hypothesized that it may influence metabolism and mROS. We found that up to 70% of the total ROS is generated by mitochondria in response to cisplatin (Fig 4), therefore, we focused on the mitochondrial related abnormalities. The antioxidant, NAC, can quench intracellular ROS that would suppress ROS-induced p53 activation (Fig 6), which is in line with previous reports [3,73]. Moreover, mitochondria host the metabolism that has been severely disrupted, so the effect of mROS on mitochondrial metabolism may provide a useful link to treat cisplatin related abnormalities. In the present study, we focused on a less appreciated prospect of cisplatin induced cell death that, despite DNA disruption, it also alters metabolism that is being orchestrated by mROS.

**Fig 7:**
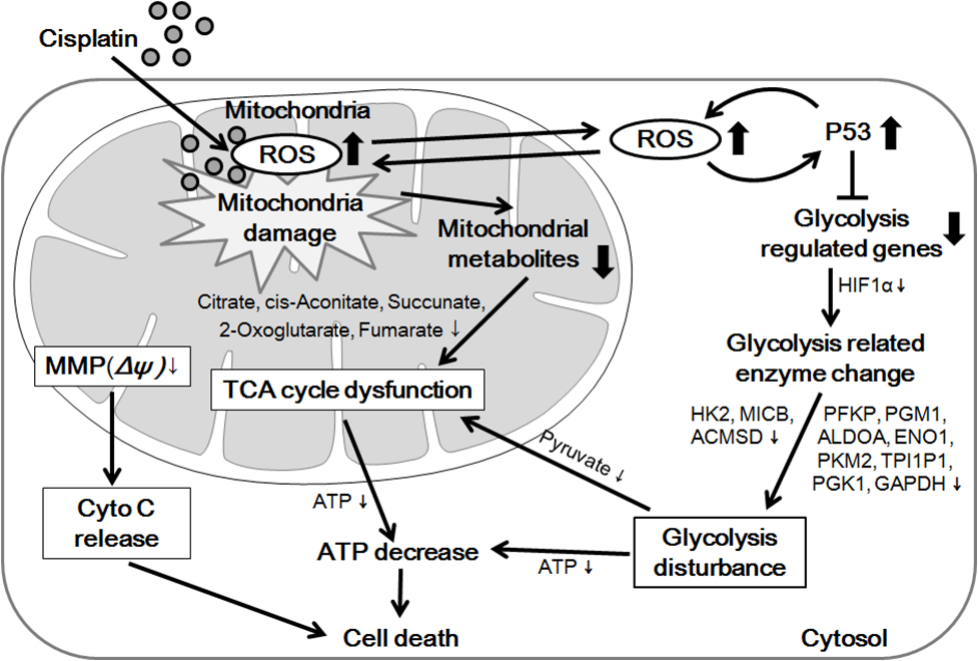
Overview of the pathway xfor cisplatin-induced metabolic dysfunction in HK-2 cells. Cisplatin induced mitochondrial dysfunction through mitochondrial reactive oxygen species (mROS) that also interfered with the tricarboxylic acid (TCA) cycle and mitochondrial stability by inhibiting the expression of enzymes and proteins essential for energy-producing pathways and the viability of mitochondria, respectively. Furthermore, the collapse of the mitochondrial membrane potential (MMP) due to mROS led to blockade of the electron-transport chain (ETC). On the other hand, mROS increased the transcription and activation of the tumor suppressor protein p53, which suppressed glycolysis and, ultimately, reduced the pyruvate level. Activated p53 also stimulated the generation of mROS, which had a synergistic effect on the functions of both, p53 and mROS. Reduction of pyruvate decreased TCA cycle functionality, which was already impaired by mROS. Therefore, cellular energy needs would not be met. Altogether, these changes result in the activation of apoptotic and necrotic pathways.

Considering the results presented here, we proposed a model for the cellular responses to the metabolic toxicity of cisplatin (Fig 7). Cisplatin accumulates in mitochondria and causes over-production of mROS, which results in mitochondrial deterioration, impaired TCA cycle, and disrupted MMP, resulting in ETC collapse. In parallel, generated mROS induces up-regulation of p53 that inhibits glycolysis, leading to the accumulation of glucose and depletion of pyruvate. The energy derived from glycolysis and mitochondrial metabolism ceases, resulting in ATP depletion. Finally, continuous ATP depletion leads to cell cycle arrest and cell death.

In conclusion, this study proposed a viable framework for cisplatin-induced metabolic dysfunction and provided a novel explanation to correlate mROS and metabolic dysfunction. It also provided the basis for a systems toxicology approach to explain mechanisms underlying toxicity effects of other drugs.

## Supporting Information

S1 Fig Labeled gene ontology analysis enrichment map. Functional analysis of transcriptomic and proteomic data was performed using Database for Annotation, Visualization, and Integrated Discovery (DAVID). Results were analyzed using the enrichment map plug-in for Cytoscape. A detailed functional network was drawn according to the identification of all labeled nodes. Fig 2 can be visualized in detail using this network.
(PDF)

S2 Fig Reconstruction of the gene and protein interaction network based on transcriptomic and proteomic analyses. After isolating metabolism-related pathways, including tricarboxylic acid (TCA) cycle/mitochondrial metabolism and glycolysis from Fig 2, a detailed map was created using STITCH and Cytoscape. Each square node indicates protein expression and each circular node indicates mRNA expression. The color of the node represents the respective fold change in the expression; red color represents up-regulation and green color represents down-regulation.
(TIF)

S3 Fig Effects of cisplatin in HK-2 cells. (A) Reactive oxygen species (ROS) production in HK-2 cells was determined after 6 and 24 h of cisplatin treatment. This was performed using the oxidant-sensitive probe 2ʹ,7ʹ-dichlorofluorescein diacetate (DCFH-DA). (B) Δψ_m_ depolarization monitored by flow cytometry with the JC-1 mitochondrial potential marker. The shift in JC-1 fluorescence from red to green indicates the collapse of the mitochondrial membrane potential (MMP). The percentage of cells with low MMP was determined.
(TIF)

S4 Fig Cisplatin induced increase in lactate and glucose concentrations that have been rescued by p53 inhibitor. (A) Glucose production in cells treated with cisplatin and 20 μM PFT-α for 24 h. Cisplatin increased the glucose concentration significantly in the cells. PFT-α has reversed the accumulation of glucose by cisplatin. (B) Lactate concentration in response to cisplatin in the presence/absence of 20 μM PFT-α for 24 h.
(TIF)

S5 Fig Ratio of fluorescence SHP-Mito dye density. (A) The fluorescence intensity of the SHP-Mito probe was measured using the imageJ program. A yellow/blue fluorescence intensity ratio of 0.63 was calculated for untreated cells; however, the yellow/blue ratio increased to 0.92 due to increased reactive oxygen species (ROS) generation in cells treated with cisplatin for 24 h. (B) Two-photon fluorescence microscopy analysis of the morphology of mitochondria stained with the SHP-Mito probe at digital high magnification. Fragmentation of mitochondria was noted in the group treated with cisplatin for 24 h (right) compared with the untreated group (left). Scale bar represents 15 μm. (C) The fluorescence intensity of the SHP-Mito probe was measured using the imageJ program. A yellow/blue fluorescence intensity ratio of 0.98 was calculated for cells treated only with cisplatin; however, the fluorescence ratio of *N*-acetylcysteine (NAC) (10 μM) and cisplatin co-treated cells decreased from 0.98 to 0.79 because mitochondrial ROS were scavenged. (D) Mitochondrial fragmentation caused by cisplatin was also reduced by NAC (10 μM) treatment when cells were observed at digital high magnification. Scale bars represent 20 μm.
(TIF)

S6 Fig Restoration of altered cell morphology and proliferation by *N*-acetylcysteine (NAC). (A) The effect of NAC (10 μM) on cisplatin-induced reactive oxygen species (ROS) production was determined with the 2ʹ,7ʹ-dichlorofluorescein diacetate (DCFH-DA) dye using flow cytometry. NAC treatment reduced the cisplatin-induced ROS generation by 1.5- to 1.1-fold compared to the untreated control. (B) HK-2 cell proliferation was assessed by the MTS assay. (C) The morphological features of HK-2 cells were assessed by phase contrast microcopy (×200). Cell viability (B) and morphology (C) results also indicated that treatment of the cells with NAC (10 μM) reduced the cytotoxic effect compared to cells treated with cisplatin alone.
(TIF)
