# Supplementary figures and images for: Mechanism of Cisplatin-Induced Cytotoxicity Is Correlated to Impaired Metabolism Due to Mitochondrial ROS Generation
Source: PLoS One. 2015 Aug 6;10(8):e0135083. doi: 10.1371/journal.pone.0135083 (PMC4527592; doi:10.1371/journal.pone.0135083)

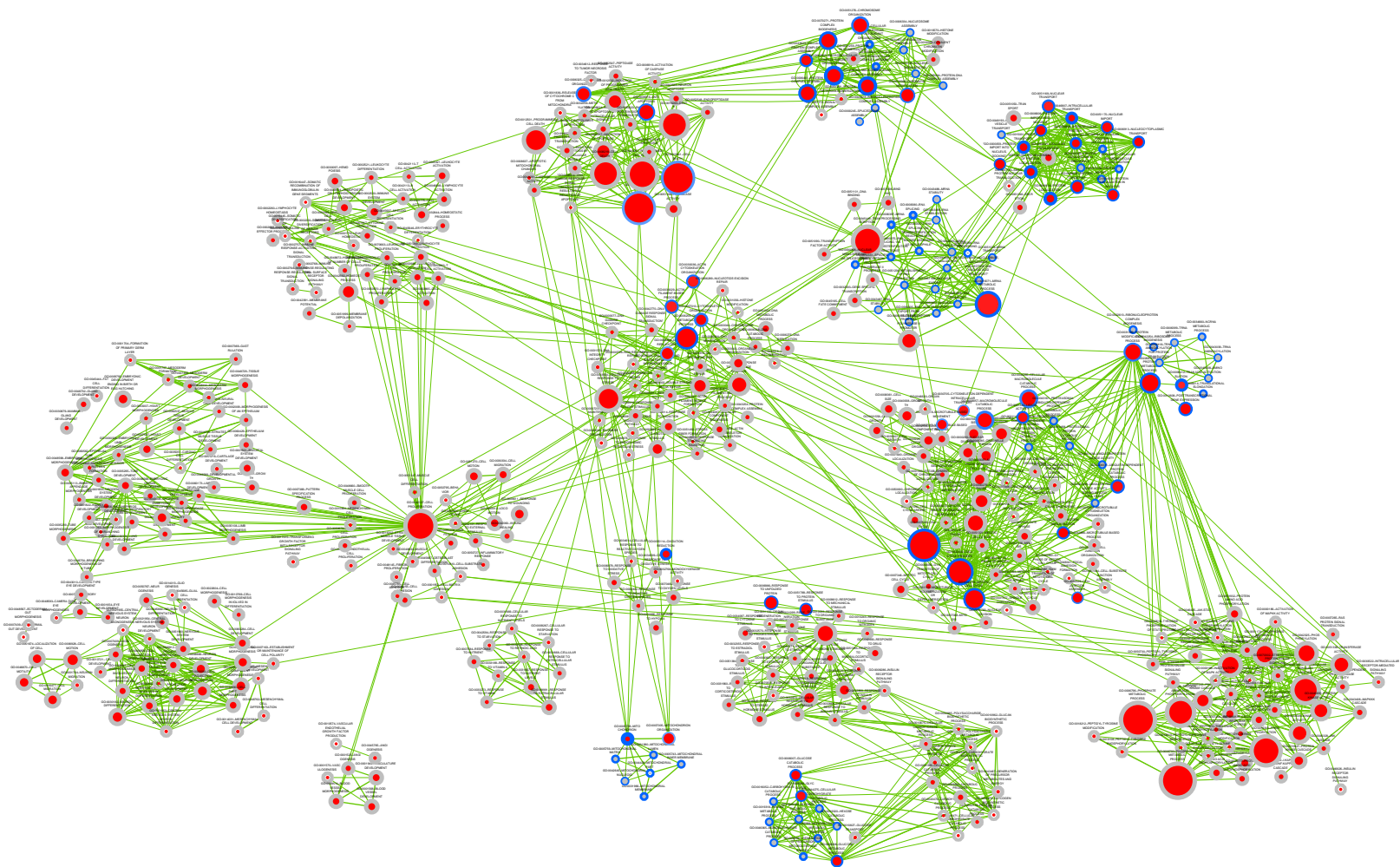

Supplement: S1 Fig — Functional analysis of transcriptomic and proteomic data was performed using Database for Annotation, Visualization, and Integrated Discovery (DAVID). Results were analyzed using the enrichment map plug-in for Cytoscape. A detailed functional network was drawn according to the identification of all labeled nodes. Fig 2 can be visualized in detail using this network. (PDF) [file pone.0135083.s001.pdf]

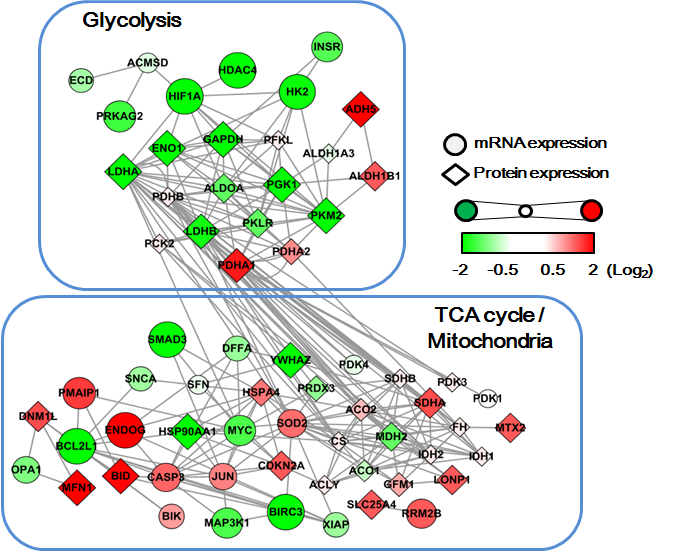

Supplement: S2 Fig — After isolating metabolism-related pathways, including tricarboxylic acid (TCA) cycle/mitochondrial metabolism and glycolysis from Fig 2, a detailed map was created using STITCH and Cytoscape. Each square node indicates protein expression and each circular node indicates mRNA expression. The color of the node represents the respective fold change in the expression; red color represents up-regulation and green color represents down-regulation. (TIF) [file pone.0135083.s002.tif]

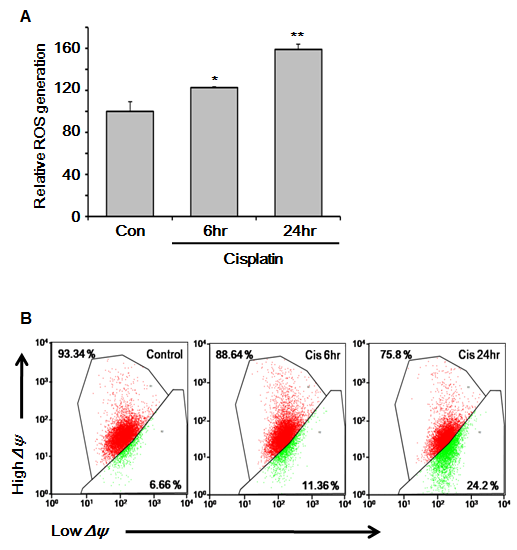

Supplement: S3 Fig — (A) Reactive oxygen species (ROS) production in HK-2 cells was determined after 6 and 24 h of cisplatin treatment. This was performed using the oxidant-sensitive probe 2ʹ,7ʹ-dichlorofluorescein diacetate (DCFH-DA). (B) Δψm depolarization monitored by flow cytometry with the JC-1 mitochondrial potential marker. The shift in JC-1 fluorescence from red to green indicates the collapse of the mitochondrial membrane potential (MMP). The percentage of cells with low MMP was determined. (TIF) [file pone.0135083.s003.tif]

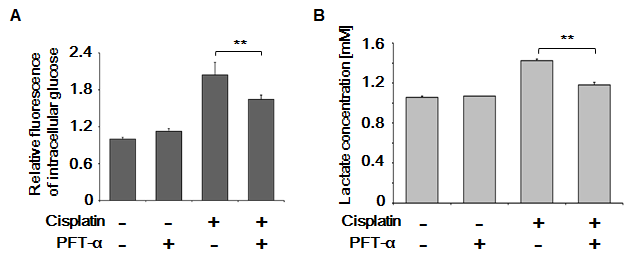

Supplement: S4 Fig — (A) Glucose production in cells treated with cisplatin and 20 μM PFT-α for 24 h. Cisplatin increased the glucose concentration significantly in the cells. PFT-α has reversed the accumulation of glucose by cisplatin. (B) Lactate concentration in response to cisplatin in the presence/absence of 20 μM PFT-α for 24 h. (TIF) [file pone.0135083.s004.tif]

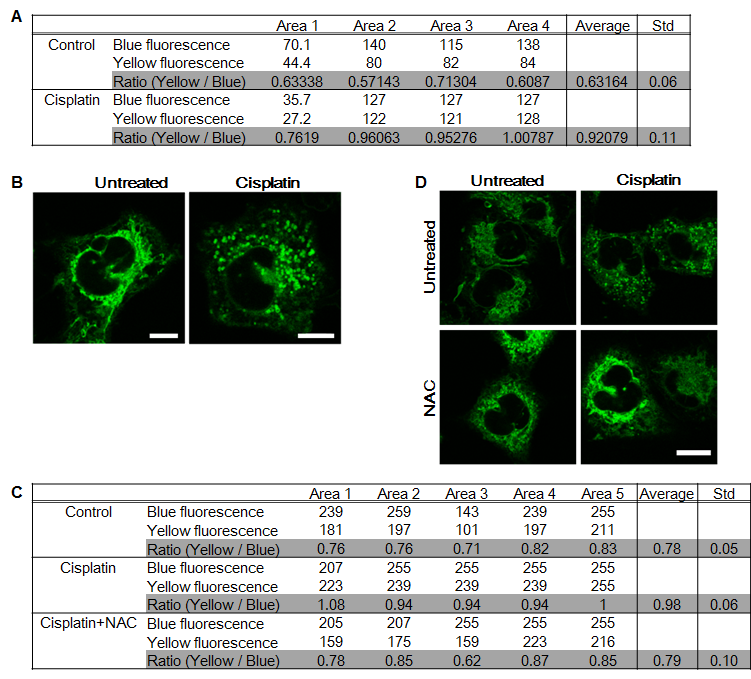

Supplement: S5 Fig — (A) The fluorescence intensity of the SHP-Mito probe was measured using the imageJ program. A yellow/blue fluorescence intensity ratio of 0.63 was calculated for untreated cells; however, the yellow/blue ratio increased to 0.92 due to increased reactive oxygen species (ROS) generation in cells treated with cisplatin for 24 h. (B) Two-photon fluorescence microscopy analysis of the morphology of mitochondria stained with the SHP-Mito probe at digital high magnification. Fragmentation of mitochondria was noted in the group treated with cisplatin for 24 h (right) compared with the untreated group (left). Scale bar represents 15 μm. (C) The fluorescence intensity of the SHP-Mito probe was measured using the imageJ program. A yellow/blue fluorescence intensity ratio of 0.98 was calculated for cells treated only with cisplatin; however, the fluorescence ratio of N-acetylcysteine (NAC) (10 μM) and cisplatin co-treated cells decreased from 0.98 to 0.79 because mitochondrial ROS were scavenged. (D) Mitochondrial fragmentation caused by cisplatin was also reduced by NAC (10 μM) treatment when cells were observed at digital high magnification. Scale bars represent 20 μm. (TIF) [file pone.0135083.s005.tif]

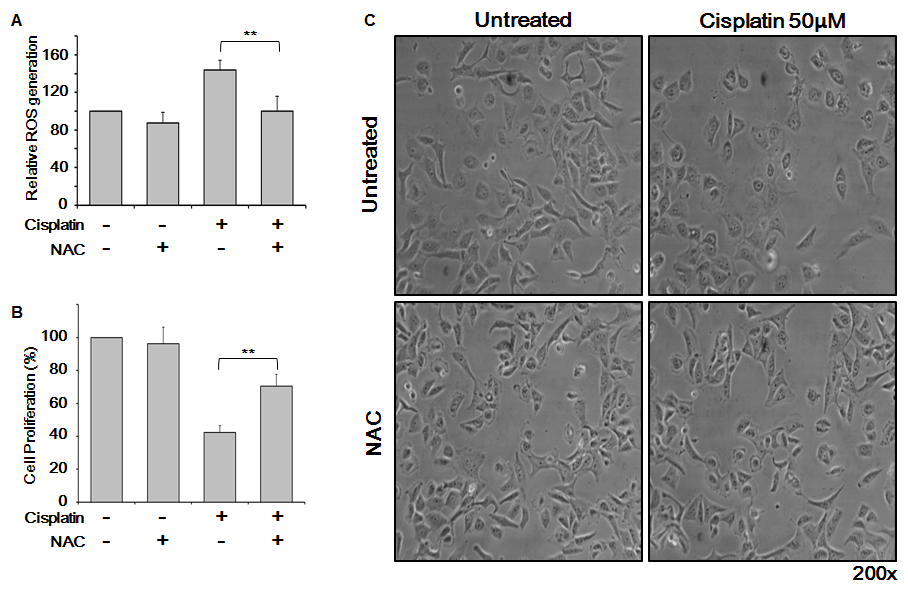

Supplement: S6 Fig — (A) The effect of NAC (10 μM) on cisplatin-induced reactive oxygen species (ROS) production was determined with the 2ʹ,7ʹ-dichlorofluorescein diacetate (DCFH-DA) dye using flow cytometry. NAC treatment reduced the cisplatin-induced ROS generation by 1.5- to 1.1-fold compared to the untreated control. (B) HK-2 cell proliferation was assessed by the MTS assay. (C) The morphological features of HK-2 cells were assessed by phase contrast microcopy (×200). Cell viability (B) and morphology (C) results also indicated that treatment of the cells with NAC (10 μM) reduced the cytotoxic effect compared to cells treated with cisplatin alone. (TIF) [file pone.0135083.s006.tif]
